# A Rare Anatomical Variation of the Termination of Right and Left Cephalic Veins

**DOI:** 10.1155/2018/5809656

**Published:** 2018-02-21

**Authors:** Raymond Saa-Eru Maalman, Yaw Otchere Donkor, Ali M. Ayamba, Jubilant Kwame Abledu

**Affiliations:** ^1^Department of Basic Medical Sciences, School of Medicine, University of Health and Allied Sciences, Ho, Volta Region, Ghana; ^2^School of Veterinary Medicine, University of Ghana, Legon, Accra, Ghana

## Abstract

Clinically, the cephalic vein is preferred for haemodialysis in patients with chronic renal failure (CRF), to remove waste products from blood. The cut-down of cephalic vein in the deltopectoral groove is preferred when superior vena caval infusion is necessary. However, cephalic veins exhibit a wide array of developmental variations in terms of formation, course, and termination. In this report, we describe a case of an anomalous cephalic vein with a bifid course of terminations on both left and right upper limbs which has not been described by previous literature. During routine gross anatomy dissection of the neck, we observed a rare case of variation of the termination of the cephalic vein in both right and left upper limbs, of a male cadaver. Knowledge of the variations of cephalic vein is important not only for anatomists but also for surgeons and clinicians as the vein is frequently used for different surgical procedures and for obtaining peripheral venous access as well.

## 1. Introduction

Cephalic vein (also called the antecubital vein) is a superficial vein of the preaxial border of the upper limb and is clearly visible in light-skinned individuals [1]. It starts as an irregular venous arch on the back of the hand and runs up along the lateral border of the forearm. It continues along the lateral border of the biceps muscle to the deltopectoral groove and perforates the clavipectoral fascia to drain into the axillary vein [2]. Clinically, the cephalic vein is preferred for haemodialysis in patients with chronic renal failure (CRF), to remove waste products from blood. The cut-down of cephalic vein in the deltopectoral groove is preferred when superior vena caval infusion is necessary. It can also be used for cardiac catheterization. It is a reliable site for cannulation when it is difficult to get access to other veins [3]. Vascular access for transvenous pacemaker and cardioverter defibrillator implants is frequently obtained using the cephalic cut-down technique which excludes the risk of pneumothorax and subclavian crush syndrome [4]. Despite the clinical importance of the cephalic vein, anatomical variations in its course and diameter of the cephalic vein may limit or complicate insertion of one or several leads. A supraclavicular course of the cephalic vein was reported in 0.2% of cases [5]. Anastasopoulos et al. [6] reported in 2% of cases the occurrence of anastomosis of cephalic vein with external juguar vein (EJV) via a communicating branch. Knowledge of these variations is essential to clinicians and surgeons for venous access during emergencies and surgery [7]. In this report, we describe a case of an anomalous cephalic vein with a bifid course of terminations on both left and right upper limbs which has not been described by the previous literature.

## 2. Case Report

**Figure 1:**
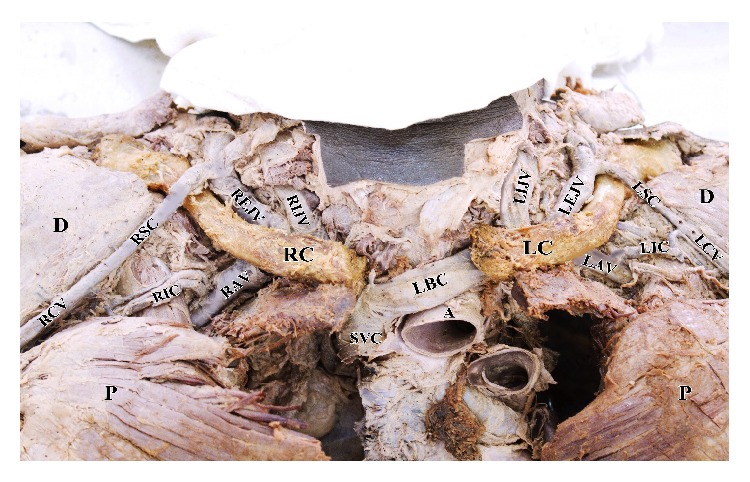
Dissected body with the clavicles (RC and LC) in situ, right and left internal jugular vein (RIJV and LIJV), right and left external jugular vein (REJV and LEJV), right and left supraclavicular (RSC and LSC) branches of cephalic vein, right and left cephalic vein (RCV and LCV), right and left infraclavicular (RIC and LIC) branch of cephalic vein, right and left axillary veins (RAV and LAV), left brachiocephalic vein (LBC), superior vena cava (SVC), aorta (A), deltoid muscle (D), and pectoral major muscle (P).

**Figure 2:**
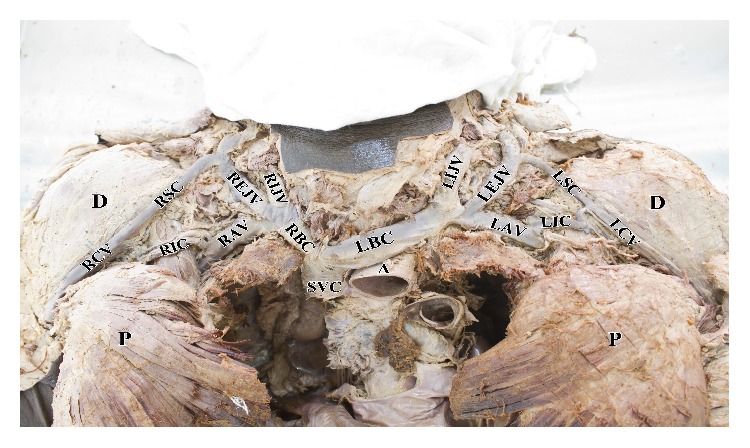
Dissected body with the clavicle removed. It shows the right and left internal jugular vein (RIJV and LIJV), right and left external jugular vein (REJV and LEJV), right and left supraclavicular (RSC and LSC) branches of cephalic vein, right and left cephalic vein (RCV and LCV), right and left infraclavicular (RIC and LIC) branch of cephalic vein, right and left axillary veins (RAV and LAV), right and left brachiocephalic vein (RBC and LBC), superior vena cava (SVC), aorta (A), deltoid muscle (D), and pectoral major muscle (P).

**Figure 3:**
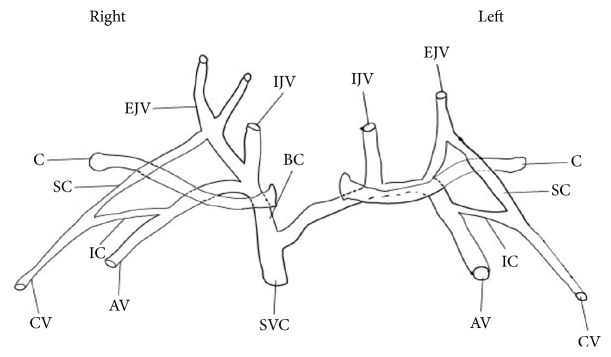
Schematic diagram showing the clavicle (C), the internal jugular vein (IJV), external jugular vein (EJV), supraclavicular (SC) branches of cephalic vein, cephalic vein (CV), infraclavicular (IC) branches of cephalic vein, axillary veins (AV), brachiocephalic vein (BC), and superior vena cava (SVC).

During routine gross anatomy dissection of the neck, we observed a rare case of variation of the termination of the cephalic vein in both right and left upper limbs, of a male cadaver. The cephalic veins of both right and left upper limbs course a supraclavicular route to drain into the right and left external jugular veins, respectively. In the deltopectoral groove, each cephalic vein gave off a smaller branch, which drains into the axillary vein. The right supraclavicular branch of the cephalic vein was observed to be larger in diameter than the left (shown in Figures 1–3). The venous architecture forms a circle of veins around each clavicle.

## 3. Discussion

In the 2nd century, AD, Galen of Pergamon transposed his observations made on monkeys onto humans and claimed that the cephalic vein (Galen's humeral vein) “arose” from the external jugular vein and encircling the clavicle “ran towards the periphery” [8]. Later studies, however, found that the cephalic vein courses in the upper arm lateral to biceps, to the deltopectoral groove, and perforates the clavipectoral fascia in the infraclavicular fossa to drain into the axillary vein [2]. Cephalic veins of the upper limbs exhibit a wide array of developmental variations in terms of formation, course, and termination [9].

In a previous study [7], two out of thirty-seven (5.4%) cases had cephalic vein crossed anteriorly and above the lateral third of the clavicle and terminated in the neck by draining directly into the external jugular vein. Loukas et al. [10] found a collateral branch between the cephalic and the external jugular vein in 2% of 200 limbs examined. However, in this case study the cephalic vein was terminated by dividing into two in the deltopectoral groove. The large branch of cephalic on each limb crossed the anterior lateral 1/3 of the clavicle to drain into external jugular vein 1 cm above the clavicle. Such a variation, developmentally corresponding to the persistent jugulocephalic vein (JCV), is typical of some nonhuman primates and is only sporadically described in humans [8]. Other studies [10, 11] have also given support to our findings that cephalic vein may ascend above the clavicle anteriorly and terminate in the neck by draining into the external jugular vein. Several other variations of the cephalic vein had been demonstrated by a case reported by Anastasopoulos et al. [6] where the cephalic vein was anastomosed with the posterior external jugular vein and not the external jugular vein.

The smaller branch of the cephalic vein in this case study was found to perforate the clavipectoral fascia in the infraclavicular fossa to drain into the axillary vein. The clavicle is thereby surrounded by venous ring made up of the larger branch of cephalic vein anteriorly, the smaller branch draining into the axillary vein inferiorly, and the external jugular vein draining into the subclavian vein superomedially. Clinically, fracture of the clavicle may injure the venous ring, resulting in severe bleeding. In addition, if cardiac catheter is to be passed in this case, an unexpected upward catheter deviation through the external jugular vein may occur during catheterization to access cardiac cavities. It may also pose difficulty in identifying the cephalic vein thereby leading to misinterpretation of the presence of cephalic vein, especially during dissections or cut-down procedure.

## 4. Conclusion

To conclude, cephalic vein termination varies and is of immense clinical importance for clinicians and surgeons as external jugular vein is commonly used for various clinical and diagnostic procedures in cardiac catheterization as well as cut-down procedures for the cephalic vein. Unexpected variation of the cephalic vein may result in inadvertent injury. This will therefore assist in identifying and locating the cephalic vein while attempting catheterization and cannulation during emergencies and surgery.
