# Corrigendum to “A Rare Anatomical Variation of the Termination of Right and Left Cephalic Veins”

**DOI:** 10.1155/2018/6735978

**Published:** 2018-05-14

**Authors:** Raymond Saa-Eru Maalman, Yaw Otchere Donkor, Ali M. Ayamba, Jubilant Kwame Abledu

**Affiliations:** ^1^Department of Basic Medical Sciences, School of Medicine, University of Health and Allied Sciences, Ho, Volta Region, Ghana; ^2^School of Veterinary Medicine, University of Ghana, Legon, Accra, Ghana

In the article titled “A Rare Anatomical Variation of the Termination of Right and Left Cephalic Veins” [1], there were errors in the Abstract and the Introduction section.

In the Abstract, the text reading “In this report, we describe a case of an anomalous cephalic vein with a bifid course of terminations on both left and right upper limbs which has not been described by previous literature” should be corrected as “In this report, we describe a case of an anomalous cephalic vein with a bifid course of terminations on both left and right upper limbs which has not been described by the previous literature.”

In addition, the text reading “During routine gross anatomy dissection of the neck, we observed a rare case of variation of the termination of the cephalic vein in both right and left upper limbs, of a male cadaver” should be removed.

In the Introduction section, the text reading “In this report, we describe a case of an anomalous cephalic vein with a bifid course of terminations on both left and right upper limbs which has not been described by the previous literature” should be removed.

In addition, the text reading “The cut-down of cephalic vein in the deltopectoral groove is preferred when superior vena caval infusion is necessary” should be removed.

Finally, the text reading “Despite the clinical importance of the cephalic vein, anatomical variations in its course and diameter of the cephalic vein may limit or complicate insertion of one or several leads” should be corrected as “Despite the clinical importance of the cephalic vein, anatomical variations in its course and diameter may limit or complicate insertion of one or several leads.”
